# Human microbiota influence the immune cell composition and gene expression in the tumor environment of a murine model of glioma

**DOI:** 10.1080/19490976.2025.2508432

**Published:** 2025-05-30

**Authors:** George B. H. Green, Alexis N. Cox-Holmes, Gillian H. Marlow, Anna Claire E. Potier, Yong Wang, Lianna Zhou, Dongquan Chen, Casey D. Morrow, Braden C. McFarland

**Affiliations:** a Department of Cell, Developmental and Integrative Biology, Heersink School of Medicine, University of Alabama at Birmingham, Birmingham, USA; b Undergraduate Cancer Biology Program, Heersink School of Medicine, University of Alabama at Birmingham, Birmingham, USA; c Department of Genetics, Heersink School of Medicine, University of Alabama at Birmingham, Birmingham, AL, USA

**Keywords:** Microbiome, glioblastoma, immunotherapy, macrophage, scRNA-seq

## Abstract

**Background:**

Immunotherapy has shown success against other cancers but not glioblastoma. Previous data has revealed that microbiota influences anti-PD-1 efficacy. We have previously found that, when using gnotobiotic mice transplanted with human fecal microbiota, the gut microbial composition influenced the response to anti-PD-1 in a mouse model of glioma. However, the role of the human microbiota in influencing the mouse immune cells in the glioma microenvironment and anti-PD-1 response was largely unknown. Using two distinct humanized microbiome (HuM) lines, we used single-cell RNA sequencing (scRNA-seq) to determine how gut microbiota affect immune infiltration and gene expression in a murine glioma model.

**Methods:**

16S rRNA sequencing was performed on fecal samples from HuM1 (H1) and HuM2 (H2) mice. Mice were intracranially injected with murine glioma cells (GL261), and on day 13 treated with one dose of isotype control or anti-PD1. Mice were euthanized on day 14 for analysis of all immune cells in the tumors by scRNA-seq.

**Results:**

HuM1 and HuM2 mice had different microbial populations, with HuM1 being primarily dominated via *Alistipes*, and HuM2 being primarily composed of *Odoribacter*. Sc-RNA-seq of the tumor immune cells revealed 21 clusters with significant differences between H1 and H2 samples with a larger population of M1 type macrophages in H1 samples. Gene expression analysis revealed higher expression of inflammatory markers in the M1 population in H2 mice treated with anti-PD-1.

**Conclusions:**

Microbial gut communities influence the presence and gene activation patterns of immune cells in the brain tumors of mice both under control (isotype) and following anti-PD-1 treatment.

## KEY POINTS

- Microbiota differences influenced numbers and activation of immune cells in a murine glioma model.
- One day after anti-PD-1 treatment, infiltrating inflammatory macrophages (MM1) increased significantly.
- HuM2 expressed larger amounts of Nos2 in MM1 cells.

## Introduction

Glioblastoma (GBM) is the most common primary malignant brain tumor, with patients having a median survival rate of 12–16 months, with aggressive treatment regimens.^1,2^ Immune checkpoint inhibitors (ICI), such as anti-programmed death ligand 1 (α-PD-1) and anti-cytotoxic T-lymphocyte associated protein 4 (α-CTLA-4), are one type of immunotherapy, which works via antibodies blocking immunosuppressive signals on T-cells, and results in an enhanced anti-tumor response.^3^ ICIs have been successful against other cancers (e.g. melanoma, gastric cancer, colorectal cancer, bladder cancer) but unfortunately not for patients with GBM.^4,5^ Interestingly, previous research has indicated that the gut microbiota (microorganisms present inside the gut) influence the efficacy of ICI in other cancers such as melanoma and lung.^6^

The microbiome influences the growth, development, and homeostasis of the host immune system.^7–9^ Microbial population resident in the host intestine influences the development and functionality of antigen presenting cells (APCs), neutrophils and other immune cell types residing the gut; however, this is not limited to the intestine, as the microbiota influences APCs and immune cells outside of the intestinal lining.^10,11^ The effect of the microbiota on immune cell development also extends to the brain as microglia have an altered morphology and downregulation of activation genes (CD86, IL-1a, Stat1, etc.) in germ-free mice.^12^ Additionally, the T-cell repertoire may be influenced via peripheral selection due to the microbiome.^13^ Previous data has shown that the host microbiota plays a role in the differentiation of T cells such as helper T (Th) 1, Th2, Th17, and regulatory T (Treg) cells.^14,15^ Previous data has revealed the microbiome influences overall immune system maturation, and homeostasis; however, the influence of the microbiome on immune homeostasis in the brain, and the tumor microenvironment has not been fully understood.^16^

Regarding the gut microbiome and GBM, there have been reports indicating the gut microbiome and associated metabolites affect glioma growth.^17–19^ Previous results from our lab revealed potential beneficial microbial populations that influence an effective ICI treatment using a humanized microbiome mouse model.^17^ Overall, these data suggests that the gut microbiota influence overall immune response to GBM and the efficacy of therapies, but the immune mechanisms are still largely unknown.^20–22^ Through utilizing a humanized microbiome (HuM) mouse model developed previously in our lab, we study how human microbial compositions influence the glioma tumor microenvironment. In this model, mice intestinal tracts have been colonized with healthy human fecal samples. We hypothesize that variations in microbial composition will lead to distinct changes in the immune profile of the tumor microenvironment. In this study, we examined GL261 murine brain tumors one day after a single dose of control (isotype) or α-PD-1 treatment in HuM mice. Through the usage of single-cell RNA-sequencing, we analyzed the immune profile and gene expression of immune cells in the tumor microenvironment, using two significantly different humanized microbiome mouse lines (HuM1 and HuM2), and how the microbiome affects the early tumor immune profile in HuM mice during control and acute immunotherapy conditions.

## Materials and methods

### Cells and reagents

GL261 cells were cultured in DMEM/F12 media supplemented with 10% FBS, penicillin/streptomycin, and L-glutamine at 37°C at 5% CO_2_ as previously described.^17^ Anti-PD-1 (InVivoMAb clone #RMP1–14) and anti-isotype control (InVivoMAb rat IgG2a clone #2A3) were from BioXcell. The flow cytometry antibodies used were CD45-APC (BioLegend, Cat No: 103112), CD11b-PE (BD Pharmingen, Cat No: 553311), CD45-PE-Cy7 (BioLegend, Cat No: 103114), and Nos2-APC (eBioscience, Cat No: 17-5920-82).

### Mice

Cecal samples were cryopreserved from two different lines of humanized microbiome (HuM) mouse breeders (HuM1 and HuM2), which were generated from healthy human donor stool samples from previously published research, and were used for fecal transplantation into gnotobiotic mice.^17,23,24^ Gnotobiotic mice were used from the UAB Gnotobiotic Core and were C56BL/6 background (10bitFoxP3.GFP.BL/6) mice.^17^ A 100–200 µL of cecal matter was given to gnotobiotic mice, via oral gavage, which resulted in two distinct humanized microbiome mouse lines (HuM1 and HuM2). The mice were bred, and the offspring were used in the experiments for the study. Experiments involving the mice was approved via the University of Alabama (UAB) Institutional Animal Care and Use Committee (#IACUC-22612 and #IACUC-22699).

### Sample preparation and high-throughput sequencing

DNA was isolated from fecal samples using the Quick DNA Fecal/Soil Microbe Miniprep (Cat# D6010, ZYMO Research), following manufacturer’s instructions. The purified DNA was assessed via the Epoch microplate spectrophotometer (BioTek Instruments) for quantification and purity assessment. Illumina MiSeq with the 250 bp paired-end kits (Illumina, Inc.) was used targeting the V4 hypervariable region of the 16S rRNA gene of bacteria for high-throughput amplicon sequencing. The resulting sequencing was demultiplexed and formatted to FASTQ. National Center for Biotechnology Information (NCBI) Sequence Read Archive (SRA) hold the raw sequence files with the following BioProject numbers: HuM1 and HuM2 mice (BioProject #PRJNA1177436). The subgroups were designated as follows: HuM1 Isotype (*n* = 3), HuM1 PD-1 (*n* = 3), and HuM2 Isotype (*n* = 3), and HuM2 PD-1 (*n* = 3).

### Taxonomic assignment and distribution

Taxonomic profiles of HuM1 and HuM2 were determined via QIIME2 (2023.5).^25^ FASTQ sequence files were submitted into QIIME2 (2023.5)^25^ using “qiime tools import” following the Cassava 1.8 paired-end demultiplexed file format (CasavaOneEightSingleLanePerSampleDirFmt). A quality check of the sequence files was achieved using “qiime demux summarize” input command. Denoising methods were used via DADA2 (q2-dada2 denoise-paired).^26^ The representative sequences were determined using q2-feature-table tabulate-seqs input. The mafft program (q2-alignment) aligned the amplicon sequence variants (ASVs)^27^ and the results were piped into the fasttree2 (q2-phylogeny), which generated the phylogeny^28^ using the default settings. Alpha diversity was generated via the Faith’s Phylogenetic Diversity,^29^ and beta diversity was generated using unweighted UniFrac,^30^ Bray–Curtis dissimilarity metrics using “core – metrics – phylogenetic” command via the “q2-diversity plugin.” Taxonomic IDs were generated utilizing q2-feature-classifier^31^ plugin via the “classify-sklearn” command against the Silva-138–99-nb-classifier.^32^ The paired-end Illumina MiSeq analysis of the V4 segment of the raw sequence files resulted in 607,183 reads after dada2 quality checking. A total of 392 observed features were identified. The most abundant phyla seen across all samples were Firmicutes, Bacteroidota, and Actinobacteria. Phylogenetic Investigation of Communities by Reconstruction of Unobserved States (PiCRUSt2, v2.5.2) was utilized to predict functional profiles of the gut microbiota, and significance was determined via ggpicrust2. To determine significant taxa, a pairwise comparison between groups was performed using a t-test, and taxa with p-values <0.05 were considered significant.

### Intracranial injections

At <6 months of age, HuM1 and HuM2 mice were injected with GL261 tumors as previously described.^33^ On day 13 post-intracranial injection, mice were randomized to receive one i.p. injection of anti-PD-1 (200 µg) or isotype control (200 µg). Male mice were used due to availability and to maintain same sex variable among all 12 mice with the microbiome and immunotherapy variables already factored in. On day 14 post-intracranial injection, mice were euthanized and the tumors harvested for single-cell RNA-sequencing.

### Single-cell tumor harvesting and sorting

Single-cell suspension from the tumors were isolated as previously described.^33^ Briefly, the tumors were minced and digested in collagenase/DNAse solution (1 mg/mL collagenase D, 50ug/mL DNAse I in RPMI) at 37°C for 30 min. Samples were filtered through a 100 μm filter and resuspended in 10 mL of FACS buffer before pelleting at 500 g for 5 min. Tumor cells were then separated over a 70%/30% discontinuous Percoll gradient at 500 g for 30 min. Cells were collected from the middle layer and washed in FACS buffer before staining for sorting. Cells were incubated with Fc block for 10 min, then incubated with hashtag antibodies (“ACCCACCAGTAAGAC”, “GGTCGAGAGCATTCA”, “CTTGCCGCATGTCAT”), CD45-APC, and Zombie Aqua fixable viability dye for 30 min at 4°C. Cells were sorted for viable CD45^+^ cells on the BD FACS ARIA SORP in the UAB Flow Cytometry and Single-Cell Core Facility into ice-cold PBS with 0.04% BSA and three samples per treatment group were pooled to increase cell counts. This was prepped on a 10 × chromium platform and libraries using single cell 3’ reagent kit v3.1 according to manufacturer’s instructions. The Raw sequence files were submitted to the National Center for Biotechnology Information (NCBI) Sequence Read Archive (SRA) under the following BioProject number HuM1 and HuM2 mice (BioProject #PRJNA1177426).

### Single cell analysis

Demultiplexing, alignment (refdata-gex-mm10–2020-A), filtering, was determined via cell ranger (v7.1.0) (source: https://support.10xgenomics.com/single-cell-gene-expression/software/pipelines/latest/what-is-cell-ranger).Cells. Expressing <200 genes were filtered out. Cells expressing <5% mitochondrial DNA was filtered out. After quality control, a total of 10,834 cells, from 12 samples were captured. Downstream analysis, Seurat v5 was used for dimension reduction and cell clustering.^34^ Raw data was imported into R as a count matrix and log-transformed using the “NormalizeData” function in the Seurat package. Principal Component Analysis (PCA) was performed, focusing on the first 20 principal components. To determine significant differences between cell clusters, we utilized “findallmarkers”, which resulted in 21 clusters. These scores measure the similarity of cells within a cluster compared to cells in other clusters.^35–37^ The cell annotations were assigned manually using the top differentially expressed genes, computational, and canonical markers. Cell proportions were calculated as the percentage of each identified immune cell type relative to the total CD45^+^ population within the tumor microenvironment. These percentages were derived following data normalization and clustering. To assess differences in cell-type abundance between groups, log-fold change analysis was performed.

### Flow cytometry and analysis

Brain tumors from 36 mice (H1-CT *n* = 8, H1-PD *n* = 10, H2-CT *n* = 8, H2-PD *n* = 10) were isolated for flow cytometry to examine Nos2 protein expression in myeloid cells (live, CD45^hi^, CD11b^+^). Cells were surface stained, fixed and permeabilized with eBioscience Intracellular Fixation & Permeabilization Buffer Set, and intracellular stained for Nos2. Samples were run on the Attune NxT and BD LSR Fortessa at the UAB Flow Cytometry Core, and the data analyzed by FlowJo software and displayed as counts and as frequency of the population (%). Count data was analyzed with Welch ANOVA and Dunnet T3 and frequency data was analyzed with a Kruskal-Wallis and Dunn’s test and graphed with GraphPad Prism.

## Results

### HuM1 and HuM2 mice have significantly different gut microbial compositions

**Figure 1.**
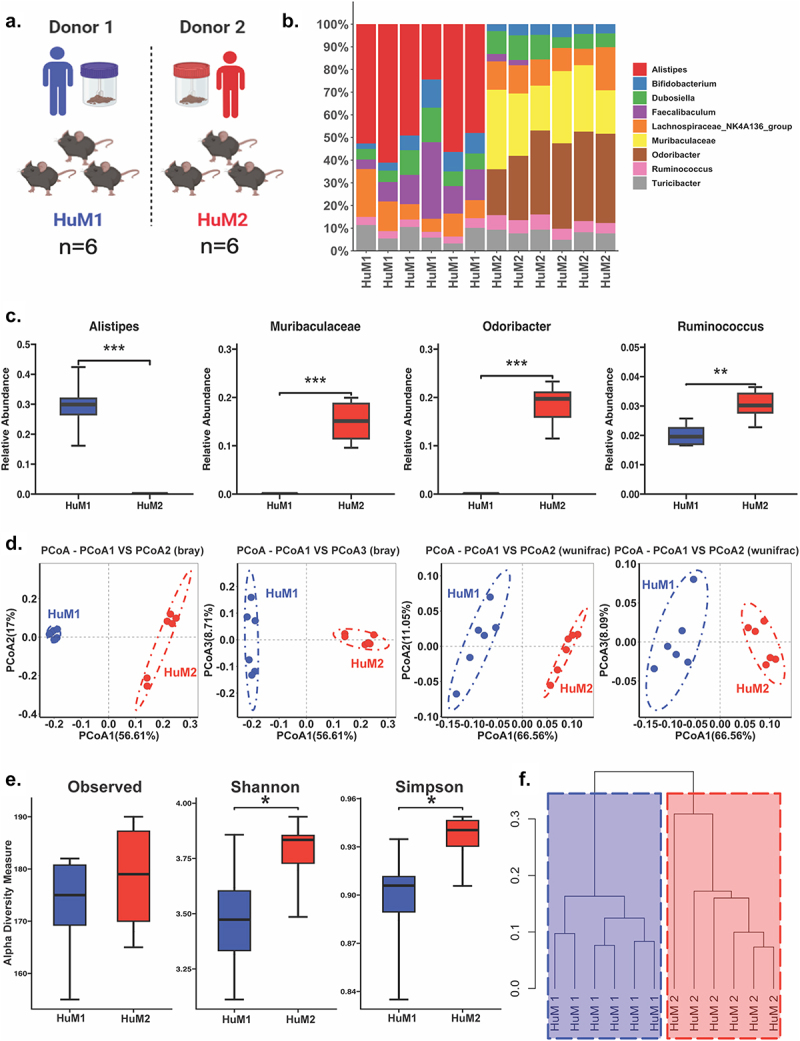
HuM1 and HuM2 are unique microbiota lines. (a) Schematic of microbiome samples. (b) The relative abundance of the top 10 taxa at the family level and genus level across HuM1 and HuM2 samples. (c) Boxplots revealing significant differences in taxa between HuM1 and HuM2. (d) Beta diversity was determined utilizing the Bray-Curtis and weighted UniFrac metrics. (e) Alpha-diversity measurements (observed ASVs, Shannon diversity index, and Simpson’s index) were determined within HuM1 and HuM2. (f) Dendrogram unique microbial compositions between HuM1 and HuM2 samples. *p < 0.01; **p < 0.01; ***p < 0.001.

To compare the composition of fecal microbe community between the HuM1 and HuM2 mice, fecal samples were collected (Figure 1a) and 16s sequencing performed for prokaryotic DNA. Taxonomic analysis revealed that HuM1 and HuM2 differed in the relative abundance of numerous microbes at the genus level (Figure 1b). Specifically, there were significantly higher levels of *Alistipes* in HuM1 mice compared to HuM2, and significantly higher levels of *Muribaculaceae*, *Odoribacter*, and *Ruminococcus* in the HuM2 mice compared to HuM1 (Figure 1c). In determining differences between groups, Beta diversity was determined utilizing Bray–Curtis and weighted UniFrac metrics across HuM1 and HuM2 mice. HuM1 and HuM2 mice displayed distinct clustering between mouse lines (Figure 1d). PERMANOVA statistics supported significant dissimilarity among sample groups (R^2^ = 0.7), with *p* values (<0.05); and PERMDISP revealed no significant dispersion of samples (*p* > 0.05). With regard to alpha diversity, or diversity within a sample, there was no significant difference in observed ASVs, but Shannon diversity and Simpson diversity revealed a significant difference between HuM1 and HuM2 groups indicating a higher alpha diversity in the HuM2 mice (Figure 1e). Lastly, a dendrogram revealed distinct separation between HuM1 and HuM2 samples (Figure 1f).

To assess the potential functional differences between HuM1 and HuM2 microbiota, PiCrust2 analyses, a bioinformatic software designed to predict functional pathways from 16s gene, was performed. For HuM1 mice, there was a significant upregulation in KEGG pathways associated with transport and catabolism, ribosome, glycan biosynthesis and metabolism, and amino acid. Of these pathways, there is a significant upregulation in steroid hormone biosynthesis, and glycosphingolipid biosynthesis–ganglio series. In contrast, HuM2 resulted in a significant upregulation of flavone and flavanol biosynthesis, atrazine degradation, and alpha-linolenic acid (Supplementary Figure S1). Collectively, these data demonstrate that HuM1 and HuM2 mice have significantly different gut microbiomes.

### Single-cell RNA sequencing analysis of immune cells in the tumor reveal distinct cell populations

**Figure 2.**
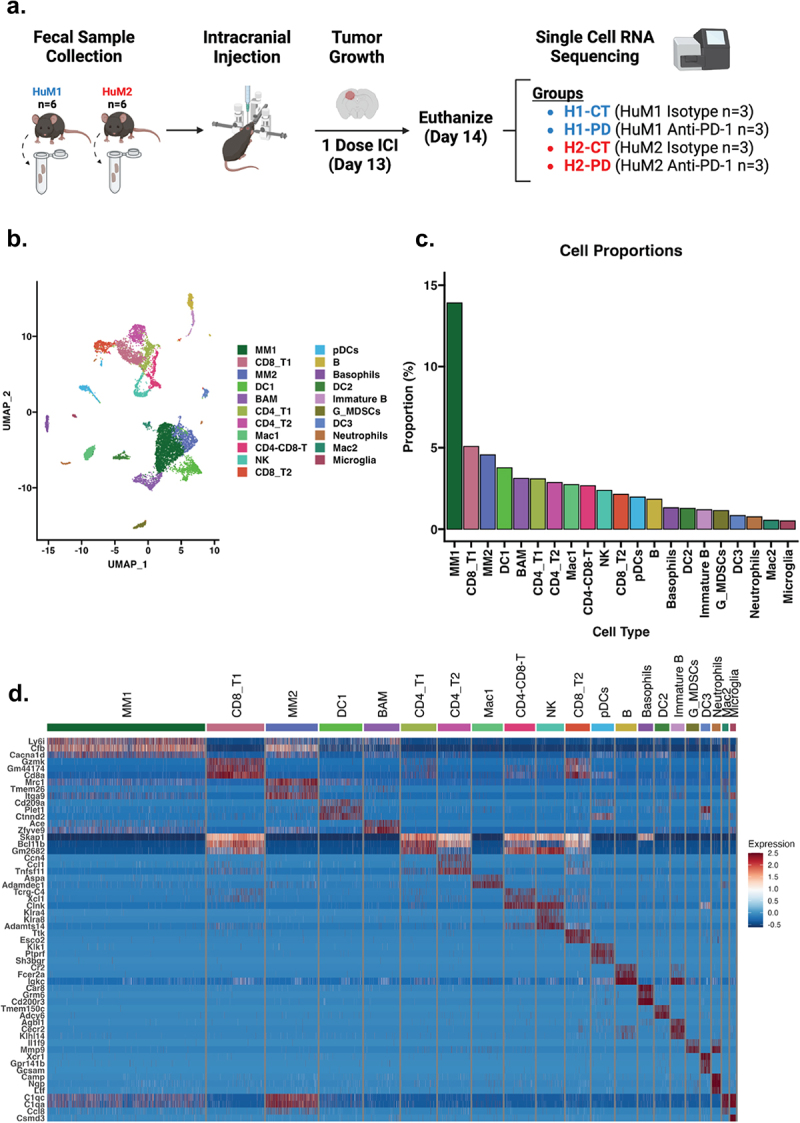
Single-cell RNA sequencing analysis of H1 and H2 samples. (a) Schematic of the single-cell experiment and sample assignments. (b) UMAP visualization of all H1 and H2 samples. Axes represent UMAP 1 and UMAP 2. (c) Bar plot showing the proportion of each cell type across samples. The y-axis represents the percentage of cells, and the x-axis represents the different sample groups. (d) Heatmap displaying the top three differentially expressed genes per cluster, ranked by average log2 Fold change, with pct.1 >0.5. The rows represent genes, and the columns correspond to cell clusters. All plots use a consistent color scheme for cell types, as indicated in the accompanying legend.

We then sought to determine if differences in gut microbiota would influence the presence or activation of immune cells in the tumor microenvironment in a mouse model of glioma that were also treated with a single dose of anti-PD-1. We injected HuM1 and HuM2 mice with GL261 cells and on day 13 treated with one dose of isotype control or anti-PD-1. We then performed single-cell RNA sequencing (scRNA-seq) on immune cells (CD45^+^) in the tumor micro-environment (Figure 2a and Supplementary Figure S2). We analyzed a total of 10,834 cells, and 18,792 features. Before splitting out each group, visualization of these cells for all groups was performed using uniform manifold approximation and projection (UMAP) (Figure 2b). A total of 10834 cells were merged into 21 clusters and were manually identified from expressed markers which were consistent without laboratories scRNA-sequencing data (Figure 2b,c). The proportion of cell numbers for each cluster reveals that the cell type that displayed the largest proportion is the MM1 population (Figure 2c). The clusters include the following: Monocyte/macrophages 1 (MM1; *Tgfbi*, *Csf1r*, *Lyz2*, *Adgre1*), CD8 T-cells (CD8_T1; *Cd8*, *Cd3*, *Cd27*, *Cd28*), Monocyte/macrophages (MM2; *Mrc1*, *Apoe*, *Csf1r*, *Tgfbi*), Dendritic cells 1 (DC1; *Dc1*, *Clec9a*, *Itgae*, *Itgax*, *Thbd*, and *Flt3*), Border associated macrophages (BAM; *Lyz2*, *Cx3cr1*, and *Adgre1*), CD4 T-cells 1 (CD4_T1; *Cd3*, *Cd4*), CD4 T-Cells (CD4_T2; *Cd4*, *Cd3d*, *Il7r*, *Ltb*, and *Trbc2*), Macrophages 1 (Mac1; *Adgre1*, *Cd80*, and *Il1a*), CD4CD8 negative T-cells (CD4-CD8-T; *Cd3d*, *Cd3g*, *Cd3e*, *Tcrg-v1*, *Tcrg-c4*, and *Tcrg-c1*), Natural killer cells (NK; *Klrb1c*, *Gzma*, and *Prf1*), CD8 T-Cell (CD8_T2; *Cd8a*, *Cd8b1*, *Ifng*, *Gzmb*, *Prf1*, *Cd3e*, and *Trbc2*), Plasmacytoid dendritic cells (pDCs; *Cd33*, *Ccr9*, *Siglech*, *Flt3*, and *Pacsin1*), B cells (*Ms4a1*, *Ighd*, *Cd19*, *Cd38*, and *Igh*), Basophils (Basophils; *Cd69*, *Cd200r3*, *Il3ra*, *Gata2*, and *Il4*), Dendritic cells 2 (DC2; *Flt3*, and *Zbtb46*), Immature B-cells (Immature B; *Cd19*, and *Cd38*), Granulocytic myeloid-derived suppressor cells (G_MDSCs; *Cd274*, *Cxcr2*, and *Cxcr4*), Dendritic cells 3 (DC3; *Flt3*, and *Zbtb46*), Neutrophils (*Cd33*, *Cxcr2*), Macrophages 2 (Mac2; *Cd163*, *Cd200r1*, and *Arg1*) and Microglia (*Tmem119*, *Cx3cr1*, and *Cd68*) (Figure 2d). A complete list of all genes is included in Supplementary Table S1.

### Significant acute increase in the influx of innate immune cells in both HuM1 and HuM2 anti-PD-1 treated mice

**Figure 3.**
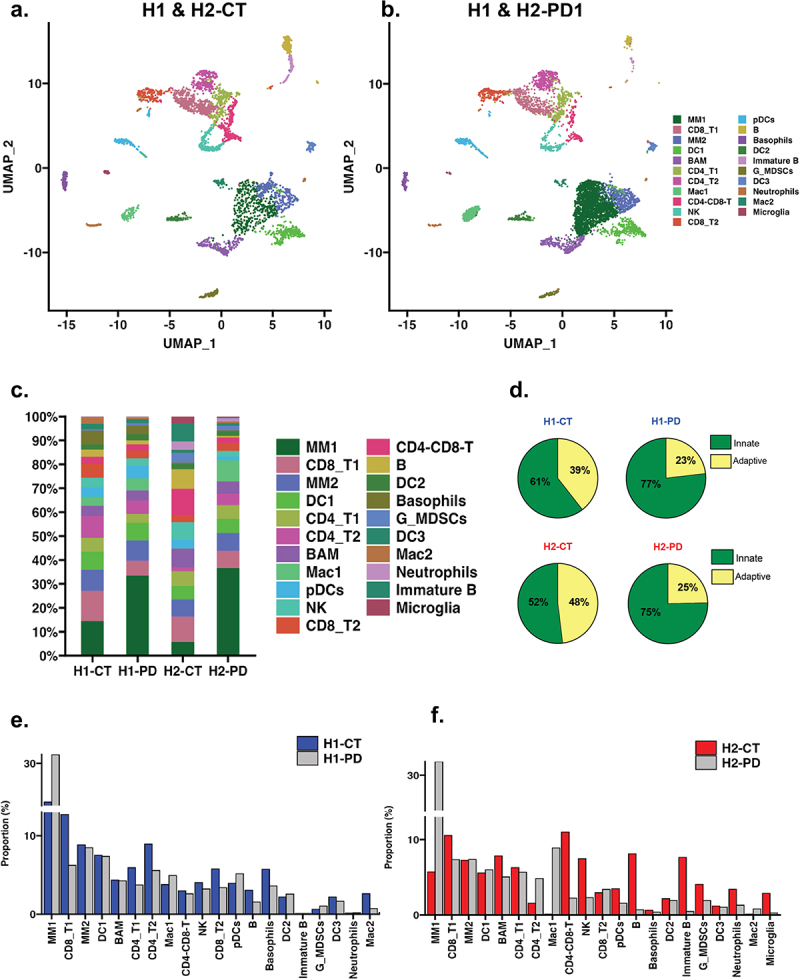
Single-cell analysis reveals differences between control and anti-PD-1 treatment samples. (a) UMAP visualization of H1 and H2 control samples. Axes represent UMAP 1 and UMAP 2. (b) UMAP visualization of H1 and H2 anti-PD-1-treated samples, with axes represent UMAP 1 and UMAP 2. (c) Stacked bar plot showing the proportion of each cell type across individual samples. The y-axis represents the percentage of cells, and the x-axis represents the sample groups. Panels (a–c) share a consistent color scheme for cell types. (d) Pie chart depicting the relative abundance of innate and adaptive immune cells across H1 and H2 samples. (e) Bar plot comparing cell type proportions between H1 control (blue) and H1 anti-PD-1-treated samples (Grey). (f) Bar plot comparing cell type proportions between H2 control (red) and H2 anti-PD-1-treated samples (Grey). Cell proportions were calculated as the percentage of each immune cell type relative to the total CD45^+^ population within the tumor microenvironment.

We first examined how one dose of anti-PD-1 treatment impacted the tumor microenvironment (including both H1 and H2 together in CT vs. PD-1). In examining the UMAP cell clusters between control and anti-PD-1 treatment, a sizable increase in the MM1 cell population was observed (Figure 3a,b). The proportion of all cell types is shown in Figure 3c, which highlights differences following anti-PD-1 for both groups. While looking at global percentages of innate versus adaptive immune cells, a substantial increase in the innate immune cell population with anti-PD-1 treatment is evident in both H1 and H2 lines (Figure 3d). The proportion changes of each cell cluster are shown for both H1 and H2, which shows the increase in MM1 population in the PD-1 treated mice is evident in both H1 and H2 groups (Figure 3e,f). This indicates that one treatment with anti-PD-1 had a strong early increase in the number of innate cells infiltrating the tumor, predominately MM1, compared to isotype treated mice regardless of microbiome status.

### In isotype control treated mice, differences in immune cells in the tumor microenvironment were found between H1 and H2 microbiome mice

**Figure 4.**
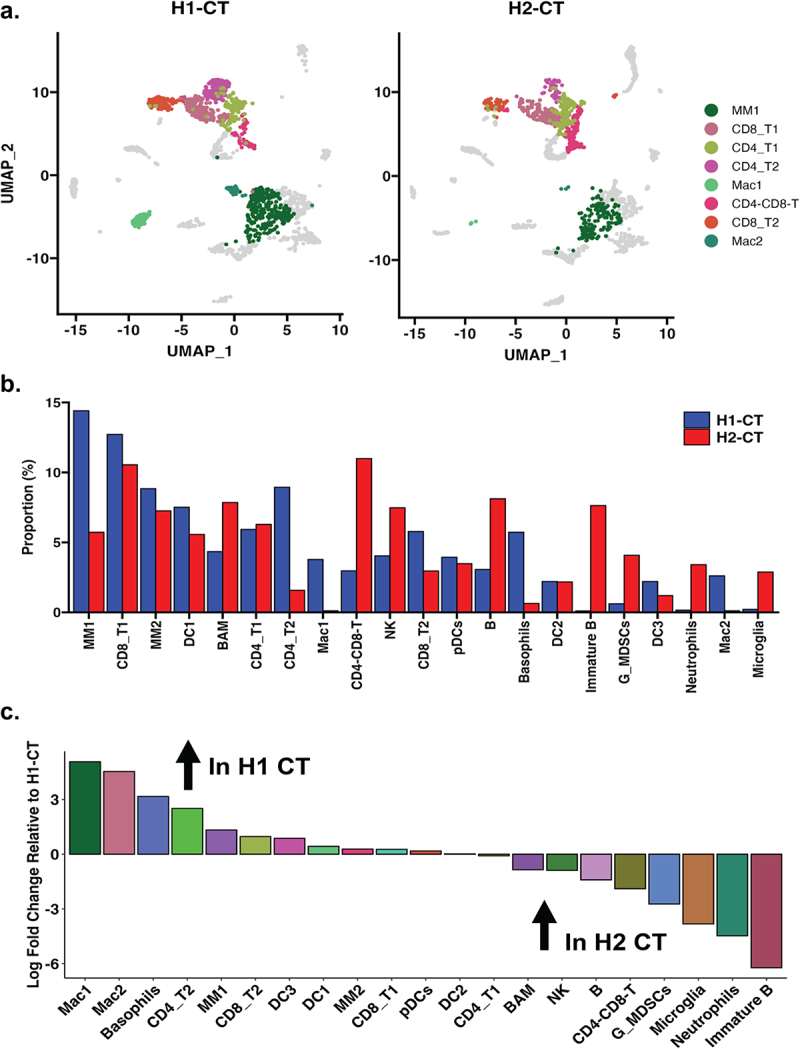
Single-cell analysis comparing H1-CT and H2-CT control samples. (a) UMAP visualization generated via Seurat (v5.1.0), highlighting T-cell and macrophage populations. Axes represent UMAP 1 and UMAP 2. (b) Bar plot showing the proportion of cell types in H1-CT (Blue) and H2-CT (red) samples. Cell proportions were calculated as the percentage of each immune cell type relative to the total CD45^+^ population within the tumor microenvironment. The y-axis represents the percentage of cells, and the x-axis represents the cell type. (c) Divergent bar plot displaying the differences in all identified cell clusters between H1-CT and H2-CT control samples.

Next, we examined the differences between treatment groups for H1 and H2 mice, starting with isotype control samples (CT). There were several differences in the proportion of immune cells in the isotype treated mice between the H1 and H2 microbiome lines. The differences between H1 and H2 CT samples were highlighted: MM1, CD8_T1, CD4_T1, CD4_T2, Mac1, CD8_T2, and Mac2 cell types (Figure 4a). Specifically, H1-CT samples had higher populations of MM1, CD8_T1, MM2, DC1, CD4_T2, Mac1, and CD8_T2. In contrast, H2-CT had an increase in BAM, CD4-CD8-T, NK, B, immature B, G_MDSCs, Neutrophils, and Microglia (Figure 4b,c). These data indicate that the gut microbiota can impact the proportions of immune cells in the tumor microenvironment in control treated conditions. In other words, the types of immune cells in the tumor environment vary depending on the gut microbiome of the mice.

### In anti-PD-1 treated mice, early differences in immune cells in the tumor microenvironment were also observed between H1 and H2 microbiome mice

**Figure 5.**
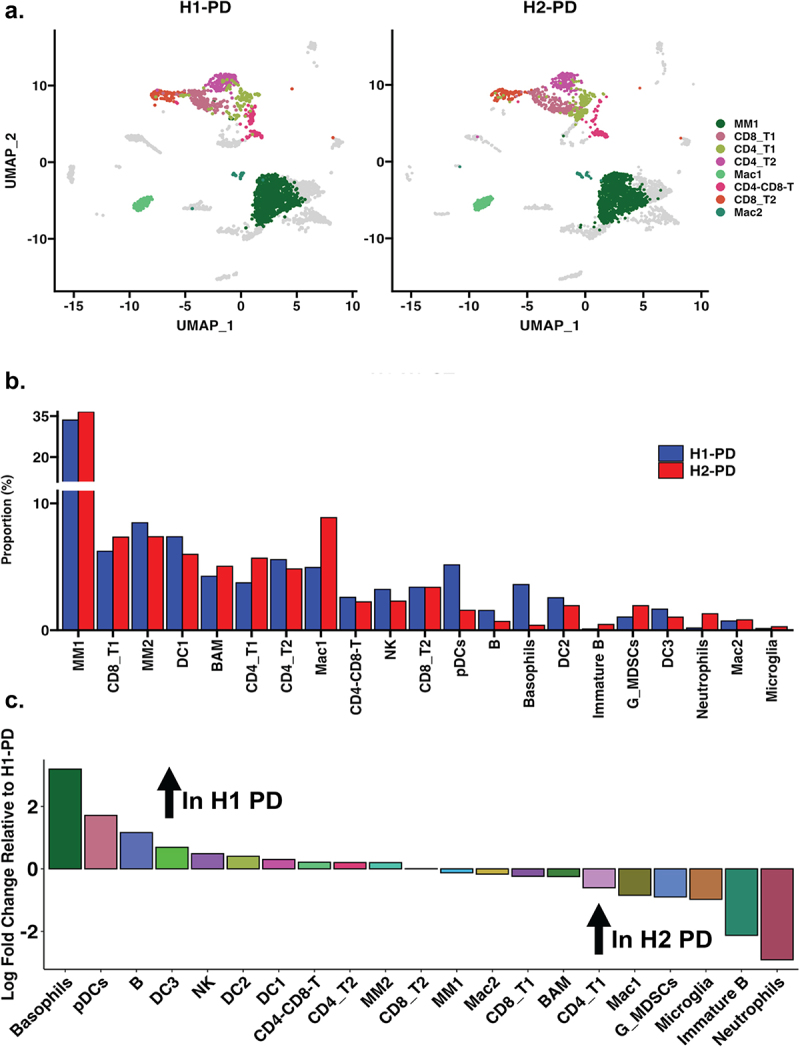
Single-cell analysis comparing H1-PD and H2-PD treatment samples. (a) UMAP visualization generated via Seurat (v5.1.0), highlighting T-cell and macrophage populations. Axes represent UMAP 1 and UMAP 2. (b) Bar plot showing the proportion of cell types in H1-PD (blue) and H2-PD (red) samples. Cell proportions were calculated as the percentage of each immune cell type relative to the total CD45^+^ population within the tumor microenvironment. The y-axis represents the percentage of cells, and the x-axis represents the cell type. (c) Divergent bar plot displaying the differences in all identified cell clusters between H1-PD and H2-PD treatment samples.

Next, we examined the differences between immune cells in anti-PD-1 groups for H1 and H2 mice. There were several differences in the proportion of immune cells in the anti-PD-1 treated mice between the H1 and H2 mice. The differences between H1 and H2 PD samples are highlighted: MM1, CD8_T1, CD4_T1, CD4_T2, Mac1, CD8_T2, and Mac2 cell types (Figure 5a). Both H1-PD and H2-PD samples were primarily dominated by the MM1 type cells. There were differences between the H1-PD and H2-PD groups, as shown by higher populations of Basophils, PDCs, B cells, in H1-PD-1, whereas in contrast H2-PD had higher abundances of Neutrophils, Immature B, and Microglia cells (Figure 5b,c). These data indicate that the gut microbiota can impact the proportions of immune cells in the tumor microenvironment one day after a single dose of anti-PD-1 therapy.

### The MM1 population in HuM2 mice display enhanced anti-tumor gene expression and reduced immunosuppressive markers

**Figure 6.**
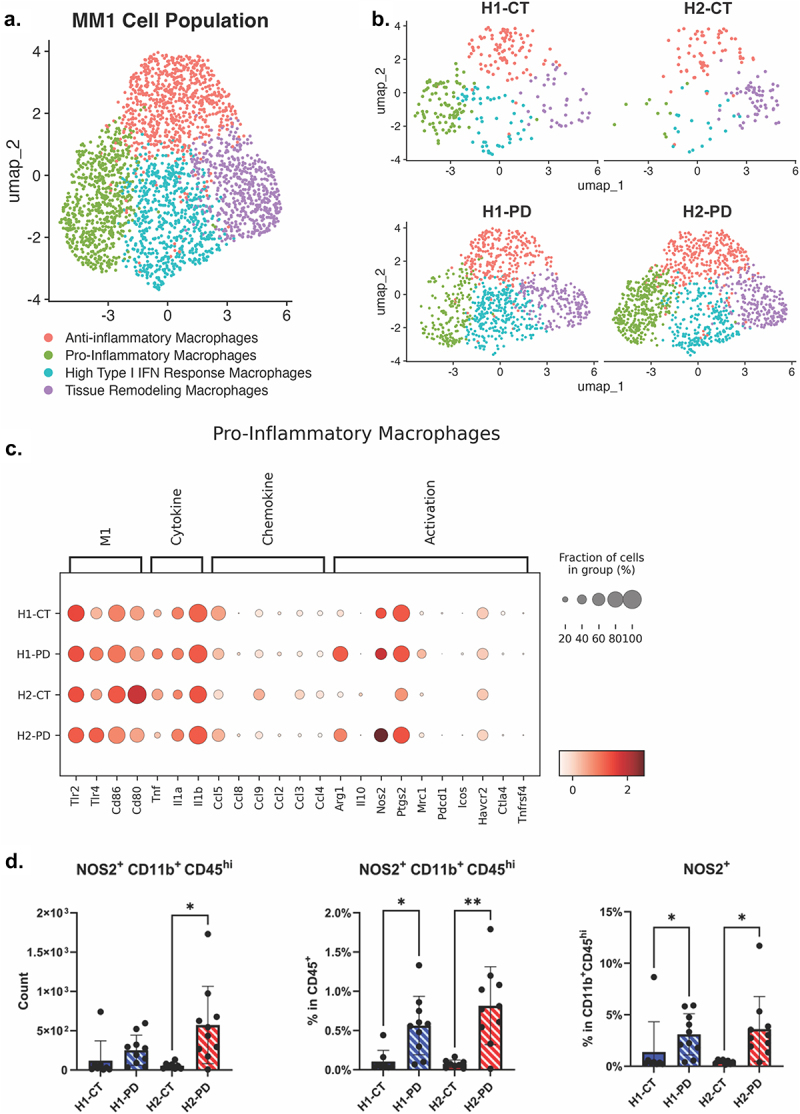
Single cell analysis of MM1 (monocytes and macrophages type 1) cell population. (a) UMAP generated, subsetting MM1 cell population. (b) UMAP generated via Seurat (v5.1.0) of MM1 separated based on samples. (c) Dot plot generated via scanpy (v1.10.2) revealing differences of MM1 cell population between all sample groups. (d) Flow cytometry revealing differences between sample groups. *p < 0.05; **p < 0.01.

Both H1 and H2 microbiome mice displayed increased percentage of MM1 population with respect to numbers in the PD-1 treated mice compared to isotype CT. Therefore, we sought to determine if there were differences in gene expression in the MM1 population among H1 and H2 groups that would predict a potential difference in phenotype or behavior *in vivo*. The MM1 cell type was more abundant in the H1-CT group compared to H2-CT; however, one day after a single anti-PD-1 treatment, both H1-PD and H2-PD groups were primarily composed of MM1 cells (Figure 6a,b). The MM1 cell population was further subsetted and re-analyzed with Seurat (v5.1.0), resulting in four different phenotypes: Anti-inflammatory Macrophages, Pro-inflammatory Macrophages, Type I IFN Response Macrophages, and Tissue Remodeling Macrophages (Figure 6a). Additionally, an acute increase in pro-inflammatory macrophages was observed in the H2-PD compared to the H1-PD, along with a noticeable increase in *Nos2* expression (Figure 6b,c). Cell markers for each are shown in Supplementary Figure S3. *Tlr2*, *Tlr4*, and *Cd86* were the markers used to identify the MM1 population and are expressed across all groups (Figure 6c). When looking at *Arg1*, an immunosuppressive marker, we found that the anti-PD-1 treated mice (both H1 and H2) displayed an increase compared to isotype (CT) groups; however, the increase was higher in the H1-PD group compared to H2-PD. *Mrc1*, the gene encoding CD206, is another immunosuppressive cell surface receptor and was lowest in the H2-PD group. Interestingly, we found that the expression of the anti-tumor inflammatory gene *Nos2* was increased in both the H1-PD and H2-PD mice, but this was more striking in the H2-PD mice (Figure 6c and Supplementary Figure S3). This indicated that the microbiota in HuM2 promotes more MM1 cells to express higher inflammatory genes and lower immunosuppressive genes in the tumor, which could be beneficial in aiding anti-tumor efforts.

To validate our findings of inflammatory markers in the MM1 population at the protein level, we injected HuM1 and HuM2 mice with GL261 cells, treated with one dose of anti-isotype control or anti-PD-1 on day 13, and all mice were euthanized the following day for analysis of immune cells by flow cytometry. We found that there was a significant increase in the NOS2^+^ CD11b^+^ CD45^+hi^ count in the H2-PD group (Figure 6d). Additionally, there were significant differences between H1-CT and H1-PD, as well as H2-CT and H2-PD in NOS2^+^ CD11b^+^CD45^+hi^ across the percentage of CD45^+^ cells as well as CD11b^+^ CD45^+hi^ cells.

Additionally, we subsetted the CD8 T cell populations, and re-analyzed with Seurat (v5.1.0). Five different phenotypes were observed: Proliferating CD8 T cells, CD8 T Resident Memory-Like (TRM-like) cells, Precursor Exhausted CD8+ T cells (Tpex), CD8 T Effector cells (Teff), and Terminally Exhausted CD8 T cell (Tex) (Supplementary Figure S4). Interestingly, there was an increased population of Tpex cells in H2-CT compared to the H2-PD group. We also found that there were fewer Tex cells in the H2-CT compared to H1-CT group. These data indicate that there could be a less exhausted T-cell phenotype in the tumors of the H2 mice compared to H1 mice.

## Discussion

The microbiome has been recognized as a significant factor in cancer development and immunotherapy.^6,38^ Our data utilized two unique microbial compositions, in which HuM1 samples were primarily dominated via *Alistipes*, whereas HuM2 is primarily dominated via members of the family Muribaculaceae. *Alistipes* may have protective effects against cancer immunotherapy; however, other studies have indicated negative effects of *Alistipes*, as Bacteroidetes members have been commonly associated with chronic intestinal inflammation.^9^ A potential contribution to immune changes with anti-PD1 therapy is that inflammation may be caused via the lipopolysaccharide (LPS) in *Alistipes*, which results in an increase in the abundance of Th17 cells expressing CD161 and CCR6/integrin Beta7. This results in a decrease in butyrate-producing bacteria, which is known for its anti-inflammatory properties.^39,40^ In colon cancer, *Alistipes* has been associated with tumor development as *Alistipes* spp. increase activation of the IL-6/STAT3 pathway and thus lead to inflammation induced tumor development.^41,42^ This IL-6 activation was MyD88-dependent so likely required toll-like receptor (TLR) family signals from the *Alistipes* spp such as LPS-TLR4 and peptidoglycan-TLR2.^43^ Muribaculaceae has been proven to contribute to the synthesis of SCFA and to be involved in mammalian longevity and antitumor effects, which was in a large abundance amongst HuM2 samples.^44^ Muribaculaceae are also known to produce B vitamins, and we observed that predicted vitamin B6 metabolism was decreased in HuM2 (Supplementary Figure S1) suggesting vitamin B6 may be increased in HuM2.^45^ Vitamin B6, or pyridoxal-5′-phosphate (PLP), is a necessary cofactor for sphingosine phosphate lyase (SPL), the enzyme that maintains the sphingosine-1-phosphate (S1P) gradient allowing immune cells to traffic between the tissues and lymph nodes.^43,46,47^ Thus, increased immune cell trafficking may be a potential mechanism behind the immune changes in HuM2 mice. Members of *Odoribacter* observed in HuM2 has been shown to exhibit anti-colorectal cancer activity via induced anti-proliferative activity via apoptosis,^48^ which may contribute to the efficacy of anti-PD1 treatment in these mice. Lastly, *Ruminococcus* was observed to be increased in the colons of HuM2 mice, and multiple studies have associated various *Ruminococcus* spp. with improved ICB outcomes. Zhang et al. (2023) found that intratumoral *Ruminococcus gnavus* degraded lyso-glycerophospholipids which prevented them from accumulating in the colon tumor microenvironment of colon cancer and suppressing T cells, thus improving the anti-tumor response.^49,50^ Similarly, predicted glycerophospholipid metabolism, which produces lyso-glycerophospholipids, was decreased in HuM2 microbiota (Supplementary Figure S1), so combined with potential degradation of the lyso-glycerophospholipids by *Ruminococcus*, this could be a potential mechanism for immune changes in these mice.

H1-CT samples had a higher abundance of MM1 cells when compared to H2-CT samples, which would indicate a preferred anti-tumor microenvironment. MM1 cells, which includes M1 type macrophages M1-like macrophages are able to activate cytotoxic T cells, via antigen presentation or production of proinflammatory cytokines, which results in IFNγ production, and T-cell proliferation; however, in the H1-PD group, a large expression was observed of *Arg1*, which leads to the production anti-inflammatory factors and inhibiting the production of pro-inflammatory factors, which results in an M2-like macrophage. M2-like macrophages release immunosuppressive chemicals, which result in blocking type 1 T helper cell immune activity, as well as an increase in T helper cell type 2 immune activity. This leads to a reduction of the control over inflammatory reactions, as well as promoting tumor cell growth, and angiogenesis. Arlauckas et al. (2018) found that anti-PD-1 therapy decreases the number of Arg1^+^ TAM while increasing Arg1^−^ TAM, and Arg1 expression is triggered via interleukin-4 and lactic acid, which are synthesized via tumor cells.^51^ Arg1 converts L-arginine into urea and L-ornithine, which results in the production of proline and polyamines crucial for cell growth and collagen synthesis in tumors. Lastly, this results in suppressing T cell activation via reducing local L-arginine levels; therefore, the lower expression of Arg1 observed in H2-PD samples may indicate an anti-tumor environment.^51^ Previous data has showed that the microbiota plays a role in the polarization of M1 and M2 macrophages as *Enterococcus faecalis*, polarized colonic macrophages toward the proinflammatory M1 phenotype in mice. Additionally, Zhou et al. found that certain short chain fatty acids (butyrate, propionate, and valerate) can promote M1 macrophage polarization in a glioma model.^52^ Alternatively, butyrate and propionate have been shown to inhibit M2 polarization through GPR43 activation and HDAC inhibition.^53^ *Odoribacter spp*. (up in HuM2) produce higher levels of butyrate than other Bacteroidales members like *Alistipes* (up in HuM1).^54^ Therefore, the microbiota likely influence the composition of immune cells present in the glioma environment.

Interestingly, H2-PD group had a higher expression of *Nos2* (Nitric oxide synthase 2). *Nos2* is utilized as a marker for the M1 type macrophage phenotype, which plays a role in inflammation and also enhances macrophage migration and survival, which results in an anti-tumor microenvironment.^55^ Nitric oxide formation occurs in M1 macrophages, which occurs with the upregulation of NOS2. This results in proinflammatory cytokines and ROS, comprising tumor necrosis factor-alpha (TNF-α), IL-1β, IL-12, and IL-23, which aids to the anti-tumor environment.^56^ We have previously shown that different HuM mouse models respond differently to anti-PD-1.^17^ Our current study supports a potential immune mechanism behind the potential positive inflammatory response to one dose of anti-PD-1 with an increase in inflammatory (Nos2^+^) macrophages in the HuM2 mice compared to HuM1 mice.

In H1-PD, there was a population of Basophils and pDCs not observed in the H2-PD group. The role of basophils in glioma biology is currently unknown; however, it has been hypothesized that GBM creates a state of immune tolerance, whereas allergy states increase immune sensitivity.^57^ Candolfi et al. (2012) suggested that IFN-α is released via activated pDCs, which may play a critical role in the antitumor effect.^58^ Additionally, there was a notable increase in neutrophil populations in H2-PD, and tumor associated neutrophils (TANs) can be polarized to an anti-tumor (N1) or pro-tumor (N2) phenotype. Transforming growth factor-β (TGFβ) has been observed to promote this pro-tumor phenotype (N2 TANs); however, IFNβ or the inhibition of TGFβ signaling promotes anti-tumor phenotype of TANs (N1 TANs).^59^ Lastly, there was a larger population of immature B cells present in H2-PD samples, which may play a suppressive or pro-inflammatory role in the glioma microenvironment; however, little is known about the role of immature B cells and the tumor glioma microenvironment.

In conclusion, these data inferred that the microbial composition may impact immunotherapy treatment, and whether the gut microbiota affects the glioma immune profile of the tumor microenvironment. From the data presented via single-cell RNA sequencing, we can see distinct differences in macrophages populations, pre immunotherapy, which may contribute with the overall efficacy of treatment. Further research is necessary to determine preferred microbial compositions for successful immunotherapy treatments for patients with GBM tumors, as well as potential beneficial metabolites produced via the microbiota; however, these data provide evidence of distinct differences in immune profiles in the tumor solely due to differences in but microbial composition. While this study provides important insights into the role of microbial communities in immune infiltration and gene expression, there are several limitations to our study, and we cannot assess the overall immunotherapy response with regard to the survival of the mice from these data. The limited cohort size, the lack of direct cause-and-effect data, and the analysis conducted only 1 d after ICI treatment is acknowledged as key limitations. Future research will include larger mouse cohorts, extended time points to investigate the durability of immune responses, and more comprehensive mechanistic studies to further validate and expand upon these findings.

## Supplementary Material

Green Supplementary Files.docx

## Data Availability

All data and code will be shared upon request via the corresponding author. The 16S rRNA raw sequence files are deposited in the National Center for Biotechnology Information (NCBI) Sequence Read Archive (SRA) under BioProject ID PRJNA1177436. The scRNA-seq datasets are deposited in the NCBI SRA under BioProject ID PRJNA1177426.

## Importance of the study

This study reveals the impact microbial composition have on the glioma tumor microenvironment, which may have translational application. Previous studies have supported that microbial composition influences the efficacy of immunotherapy. A novel humanized mouse microbiome model was used to observe two distinctly different microbial compositions and how these microbes may affect the glioma tumor microenvironment. These data indicate that microbial composition affects immune cell populations, including pro- and anti-inflammatory expression markers observed in innate and adaptive immune cells. The microbiota influence both innate and adaptive cell populations, which could provide potential translation to clinical application, as modulating the microbiome with prebiotics, probiotics, or synbiotics may improve the efficacy of immunotherapy.

## References

[1] Shah S. Novel therapies in glioblastoma treatment: review of Glioblastoma; current treatment Options; and novel oncolytic viral therapies. Med Sci. 2024;12(1):1. doi: 10.3390/medsci12010001. PMC10801585 38249077

[2] Louis DN, Perry A, Wesseling P, Brat DJ, Cree IA, Figarella-Branger D, Hawkins C, Ng HK, Pfister SM, Reifenberger G, et al. The 2021 WHO classification of tumors of the central nervous system: a summary. Neuro Oncol. 2021;23(8):1231–18. doi: 10.1093/neuonc/noab106. 34185076 PMC8328013

[3] Sampson JH, Gunn MD, Fecci PE, Ashley DM. Brain immunology and immunotherapy in brain tumours. Nat Rev Cancer. 2020;20(1):12–25. doi: 10.1038/s41568-019-0224-7. 31806885 PMC7327710

[4] Lim M, Weller M, Idbaih A, Steinbach J, Finocchiaro G, Raval RR, Ansstas G, Baehring J, Taylor JW, Honnorat J, et al. Phase III trial of chemoradiotherapy with temozolomide plus nivolumab or placebo for newly diagnosed glioblastoma with methylated MGMT promoter. Neuro Oncol. 2022;24(11):1935–1949. doi: 10.1093/neuonc/noac116. 35511454 PMC9629431

[5] Omuro A, Brandes AA, Carpentier AF, Idbaih A, Reardon DA, Cloughesy T, Sumrall A, Baehring J, van den Bent M, Bähr O, et al. Radiotherapy combined with nivolumab or temozolomide for newly diagnosed glioblastoma with unmethylated MGMT promoter: an international randomized phase III trial. Neuro Oncol. 2023;25(1):123–134. doi: 10.1093/neuonc/noac099. 35419607 PMC9825306

[6] Jobin C. Precision medicine using microbiota. Science. 2018;359(6371):32–34. doi: 10.1126/science.aar2946. 29302001

[7] Wu HJ, Wu E. The role of gut microbiota in immune homeostasis and autoimmunity. Gut Microbes. 2012;3(1):4–14. doi: 10.4161/gmic.19320. 22356853 PMC3337124

[8] Lambring CB, Siraj S, Patel K, Sankpal UT, Mathew S, Basha R. Impact of the microbiome on the immune system. Crit Rev Immunol. 2019;39(5):313–328. doi: 10.1615/CritRevImmunol.2019033233. 32422014 PMC7362776

[9] Belkaid Y, Hand TW. Role of the microbiota in immunity and inflammation. Cell. 2014;157(1):121–141. doi: 10.1016/j.cell.2014.03.011. 24679531 PMC4056765

[10] Zheng D, Liwinski T, Elinav E. Interaction between microbiota and immunity in health and disease. Cell Res. 2020;30(6):492–506. doi: 10.1038/s41422-020-0332-7. 32433595 PMC7264227

[11] Zhang D, Frenette PS. Cross talk between neutrophils and the microbiota. Blood. 2019;133(20):2168–2177. doi: 10.1182/blood-2018-11-844555. 30898860 PMC6524562

[12] Erny D, Hrabě de Angelis AL, Jaitin D, Wieghofer P, Staszewski O, David E, Keren-Shaul H, Mahlakoiv T, Jakobshagen K, Buch T, et al. Host microbiota constantly control maturation and function of microglia in the CNS. Nat Neurosci. 2015;18(7):965–977. doi: 10.1038/nn.4030. 26030851 PMC5528863

[13] Shim JA, Ryu JH, Jo Y, Hong C. The role of gut microbiota in T cell immunity and immune mediated disorders. Int J Biol Sci. 2023;19(4):1178–1191. doi: 10.7150/ijbs.79430. 36923929 PMC10008692

[14] Choden T, Cohen NA. The gut microbiome and the immune system. Exploration Med. 2022;3(3):219–233. doi: 10.37349/emed.2022.00087.

[15] Shi N, Li N, Duan X, Niu H. Interaction between the gut microbiome and mucosal immune system. Mil Med Res. 2017;4(1):14. doi: 10.1186/s40779-017-0122-9. 28465831 PMC5408367

[16] Tibbs TN, Lopez LR, Arthur JC. The influence of the microbiota on immune development, chronic inflammation, and cancer in the context of aging. Microb Cell. 2019;6(8):324–334. doi: 10.15698/mic2019.08.685. 31403049 PMC6685047

[17] Dees KJ, Koo H, Humphreys JF, Hakim JA, Crossman DK, Crowley MR, Nabors LB, Benveniste EN, Morrow CD, McFarland BC. Human gut microbial communities dictate efficacy of anti-PD-1 therapy in a humanized microbiome mouse model of glioma. Neurooncol Adv. 2021;3(1). doi: 10.1093/noajnl/vdab023. PMC7967908 33758825

[18] Dono A, Nickles J, Rodriguez-Armendariz AG, McFarland BC, Ajami NJ, Ballester LY, Wargo JA, Esquenazi Y. Glioma and the gut–brain axis: opportunities and future perspectives. Neurooncol Adv. 2022;4(1):vdac054. doi: 10.1093/noajnl/vdac054. 35591978 PMC9113089

[19] D’Alessandro G, Antonangeli F, Marrocco F, Porzia A, Lauro C, Santoni A, Limatola C. Gut microbiota alterations affect glioma growth and innate immune cells involved in tumor immunosurveillance in mice. Eur. J. Immunol. 2020;50(5):705–711. doi: 10.1002/eji.201948354. 32034922 PMC7216943

[20] Zhang M, Liu J, Xia Q. Role of gut microbiome in cancer immunotherapy: from predictive biomarker to therapeutic target. Exp Hematol & Oncol. 2023;12(1):84. doi: 10.1186/s40164-023-00442-x. PMC10537950 37770953

[21] Li X, Zhang S, Guo G, Han J, Yu J. Gut microbiome in modulating immune checkpoint inhibitors. eBiomedicine. 2022;82:104163. doi: 10.1016/j.ebiom.2022.104163. 35841869 PMC9297075

[22] Lu Y, Yuan X, Wang M, He Z, Li H, Wang J, Li Q. Gut microbiota influence immunotherapy responses: mechanisms and therapeutic strategies. J Hematol & Oncol. 2022;15(1):47. doi: 10.1186/s13045-022-01273-9. 35488243 PMC9052532

[23] Koo H, McFarland BC, Hakim JA, Crossman DK, Crowley MR, Rodriguez JM, Benveniste EN, Morrow CD. An individualized mosaic of maternal microbial strains is transmitted to the infant gut microbial community. R Soc Open Sci. 2020;7(4):192200. doi: 10.1098/rsos.192200. 32431894 PMC7211887

[24] Koo H, Morrow CD. Incongruence between dominant commensal donor microbes in recipient feces post fecal transplant and response to anti-PD-1 immunotherapy. BMC Microbiol. 2021;21(1):251. doi: 10.1186/s12866-021-02312-0. 34544375 PMC8454007

[25] Bolyen E, Rideout JR, Dillon MR, Bokulich NA, Abnet CC, Al-Ghalith GA, Alexander H, Alm EJ, Arumugam M, Asnicar F, et al. Reproducible, interactive, scalable and extensible microbiome data science using QIIME 2. Nat Biotechnol. 2019;37(8):852–857. doi: 10.1038/s41587-019-0209-9. 31341288 PMC7015180

[26] Callahan BJ, McMurdie PJ, Rosen MJ, Han AW, Johnson AJA, Holmes SP. DADA2: high-resolution sample inference from illumina amplicon data. Nat Methods. 2016;13(7):581–583. doi: 10.1038/nmeth.3869. 27214047 PMC4927377

[27] Katoh K, Standley DM. MAFFT multiple sequence alignment software version 7: improvements in performance and usability. Mol Biol And Evol. 2013;30(4):772–780. doi: 10.1093/molbev/mst010. 23329690 PMC3603318

[28] Price MN, Dehal PS, Arkin AP. FastTree: computing large minimum evolution trees with profiles instead of a distance matrix. Mol Biol Evol. 2009;26(7):1641–1650. doi: 10.1093/molbev/msp077. 19377059 PMC2693737

[29] Faith DP. Conservation evaluation and phylogenetic diversity. Biol Conserv. 1992;61(1):1–10. doi: 10.1016/0006-3207(92)91201-3.

[30] Lozupone C, Lladser ME, Knights D, Stombaugh J, Knight R. UniFrac: an effective distance metric for microbial community comparison. ISME J. 2011;5(2):169–172. doi: 10.1038/ismej.2010.133. 20827291 PMC3105689

[31] Bokulich NA, Kaehler BD, Rideout JR, Dillon M, Bolyen E, Knight R, Huttley GA, Gregory Caporaso J. Optimizing taxonomic classification of marker-gene amplicon sequences with QIIME 2’s q2-feature-classifier plugin. Microbiome. 2018;6(1):1–17. doi: 10.1186/s40168-018-0470-z. 29773078 PMC5956843

[32] Yilmaz P, Parfrey LW, Yarza P, Gerken J, Pruesse E, Quast C, Schweer T, Peplies J, Ludwig W, Glöckner FO. The SILVA and “all-species living tree project (LTP)” taxonomic frameworks. Nucleic Acids Res. 2014;42(D1):D643–D648. doi: 10.1093/nar/gkt1209. 24293649 PMC3965112

[33] McFarland BC, Marks MP, Rowse AL, Fehling SC, Gerigk M, Qin H, Benveniste EN. Loss of SOCS3 in myeloid cells prolongs survival in a syngeneic model of glioma. Oncotarget. 2016;7(15):20621–20635. doi: 10.18632/oncotarget.7992. 26967393 PMC4991480

[34] Satija R, Farrell JA, Gennert D, Schier AF, Regev A. Spatial reconstruction of single-cell gene expression data. Nat Biotechnol. 2015;33(5):495–502. doi: 10.1038/nbt.3192. 25867923 PMC4430369

[35] Stuart T, Butler A, Hoffman P, Hafemeister C, Papalexi E, Mauck WM, Hao Y, Stoeckius M, Smibert P, Satija R. Comprehensive integration of single-cell data. Cell. 2019;177(7):1888–1902.e21. doi: 10.1016/j.cell.2019.05.031. 31178118 PMC6687398

[36] Hao Y, Hao S, Andersen-Nissen E, Mauck WM, Zheng S, Butler A, Lee MJ, Wilk AJ, Darby C, Zager M, et al. Integrated analysis of multimodal single-cell data. Cell. 2021;184(13):3573–3587.e29. doi: 10.1016/j.cell.2021.04.048. 34062119 PMC8238499

[37] Hao Y, Stuart T, Kowalski MH, Choudhary S, Hoffman P, Hartman A, Srivastava A, Molla G, Madad S, Fernandez-Granda C, et al. Dictionary learning for integrative, multimodal and scalable single-cell analysis. Nat Biotechnol. 2024;42(2):293–304. doi: 10.1038/s41587-023-01767-y. 37231261 PMC10928517

[38] Bessell CA, Isser A, Havel JJ, Lee S, Bell DR, Hickey JW, Chaisawangwong W, Glick Bieler J, Srivastava R, Kuo F, et al. Commensal bacteria stimulate antitumor responses via T cell cross-reactivity. JCI Insight. 2020;5(8). doi: 10.1172/jci.insight.135597. PMC7205429 32324171

[39] Jama HA, Beale A, Shihata WA, Marques FZ. The effect of diet on hypertensive pathology: is there a link via gut microbiota-driven immunometabolism? Cardiovasc Res. 2019;115(9):1435–1447. doi: 10.1093/cvr/cvz091. 30951169

[40] Parker BJ, Wearsch PA, Veloo ACM, Rodriguez-Palacios A. The genus alistipes: gut bacteria with emerging implications to inflammation, cancer, and mental health. Front Immunol. 2020;11:906. doi: 10.3389/fimmu.2020.00906. 32582143 PMC7296073

[41] Fang Y, Yan C, Zhao Q, Zhao B, Liao Y, Chen Y, Wang D, Tang D. The association between gut microbiota, toll-like receptors, and colorectal cancer. Clin Med Insights Oncol. 2022;16:11795549221130549. doi: 10.1177/11795549221130549. 36338264 PMC9634190

[42] Moschen AR, Gerner RR, Wang J, Klepsch V, Adolph T, Reider S, Hackl H, Pfister A, Schilling J, Moser P, et al. Lipocalin 2 protects from inflammation and tumorigenesis associated with gut microbiota alterations. Cell Host & Microbe. 2016;19(4):455–469. doi: 10.1016/j.chom.2016.03.007. 27078067

[43] Kuhn KA, Schulz HM, Regner EH, Severs EL, Hendrickson JD, Mehta G, Whitney AK, Ir D, Ohri N, Robertson CE, et al. Bacteroidales recruit IL-6-producing intraepithelial lymphocytes in the colon to promote barrier integrity. Mucosal Immunol. 2018;11(2):357–368. doi: 10.1038/mi.2017.55. 28812548 PMC5815964

[44] Sibai M, Altuntaş E, Yıldırım B, Öztürk G, Yıldırım S, Demircan T. Microbiome and longevity: high abundance of longevity-linked muribaculaceae in the gut of the long-living rodent spalax leucodon. Omics: A J Intgr Biol. 2020;24(10):592–601. doi: 10.1089/omi.2020.0116. 32907488

[45] Zhu Y, Chen B, Zhang X, Akbar MT, Wu T, Zhang Y, Zhi L, Shen Q. Exploration of the muribaculaceae family in the gut microbiota: diversity, metabolism, and function. Nutrients. 2024;16(16):2660. doi: 10.3390/nu16162660. 39203797 PMC11356848

[46] Zhao P, Liu ID, Hodgin JB, Benke PI, Selva J, Torta F, Wenk MR, Endrizzi JA, West O, Ou W, et al. Responsiveness of sphingosine phosphate lyase insufficiency syndrome to vitamin B6 cofactor supplementation. J Inherit Metab Dis. 2020;43(5):1131–1142. doi: 10.1002/jimd.12238. 32233035 PMC8072405

[47] Schwab SR, Pereira JP, Matloubian M, Xu Y, Huang Y, Cyster JG. Lymphocyte sequestration through S1P lyase inhibition and disruption of S1P gradients. Science. 2005;309(5741):1735–1739. doi: 10.1126/science.1113640. 16151014

[48] Oh BS, Choi WJ, Kim JS, Ryu SW, Yu SY, Lee J-S, Park S-H, Kang SW, Lee J, Jung WY, et al. Cell-free supernatant of Odoribacter splanchnicus isolated from human feces exhibits Anti-colorectal cancer activity. Front Microbiol. 2021;12:736343. doi: 10.3389/fmicb.2021.736343. 34867852 PMC8638082

[49] Di Luccia B, Molgora M, Khantakova D, Jaeger N, Chang H-W, Czepielewski RS, Helmink BA, Onufer EJ, Fachi JL, Bhattarai B, et al. TREM2 deficiency reprograms intestinal macrophages and microbiota to enhance anti–PD-1 tumor immunotherapy. Sci Immunol. 2024;9(95):eadi5374. doi: 10.1126/sciimmunol.adi5374. 38758808 PMC11299520

[50] Zhang X, Yu D, Wu D, Gao X, Shao F, Zhao M, Wang J, Ma J, Wang W, Qin X, et al. Tissue-resident lachnospiraceae family bacteria protect against colorectal carcinogenesis by promoting tumor immune surveillance. Cell Host & Microbe. 2023;31(3):418–432.e8. doi: 10.1016/j.chom.2023.01.013. 36893736

[51] Arlauckas SP, Garren SB, Garris CS, Kohler RH, Oh J, Pittet MJ, Weissleder R. Arg1 expression defines immunosuppressive subsets of tumor-associated macrophages. Theranostics. 2018;8(21):5842–5854. doi: 10.7150/thno.26888. 30613266 PMC6299430

[52] Zhou M, Wu J, Shao Y, Zhang J, Zheng R, Shi Q, Wang J, Liu B. Short-chain fatty acids reverses gut microbiota dysbiosis-promoted progression of glioblastoma by up-regulating M1 polarization in the tumor microenvironment. Int Immunopharmacol. 2024;141:112881. doi: 10.1016/j.intimp.2024.112881. 39159556

[53] Huang C, Du W, Ni Y, Lan G, Shi G. The effect of short-chain fatty acids on M2 macrophages polarization in vitro and in vivo. Clin Exp Immunol. 2022;207(1):53–64. doi: 10.1093/cei/uxab028. 35020860 PMC8802183

[54] Zhang ZJ, Cole CG, Coyne MJ, Lin H, Dylla N, Smith RC, Pappas TE, Townson SA, Laliwala N, Waligurski E, et al. Comprehensive analyses of a large human gut Bacteroidales culture collection reveal species- and strain-level diversity and evolution. Cell Host & Microbe. 2024;32(10):1853–1867.e5. doi: 10.1016/j.chom.2024.08.016. 39293438 PMC11466702

[55] Wang X, Gray Z, Willette-Brown J, Zhu F, Shi G, Jiang Q, Song N-Y, Dong L, Hu Y. Macrophage inducible nitric oxide synthase circulates inflammation and promotes lung carcinogenesis. Cell Death Discov. 2018;4(1):46. doi: 10.1038/s41420-018-0046-5. PMC5967330 29844930

[56] Wang G, Zhong K, Wang Z, Zhang Z, Tang X, Tong A, Zhou L. Tumor-associated microglia and macrophages in glioblastoma: from basic insights to therapeutic opportunities. Front Immunol. 2022;13. doi: 10.3389/fimmu.2022.964898. PMC9363573 35967394

[57] Madhugiri VS, Venkatesan S, Dutt A, Moiyadi AV, Shetty P, Gupta T, Epari S, Jalali R, Sasidharan GM, Kumar VRR, et al. An analysis of eosinophil- and basophil-based indices in patients with glioblastoma and their correlation with survival. World Neurosurg. 2023;170:e292–e300. doi: 10.1016/j.wneu.2022.11.008. 36368458

[58] Candolfi M, King GD, Yagiz K, Curtin JF, Mineharu Y, Muhammad AG, Foulad D, Kroeger KM, Barnett N, Josien R, et al. Plasmacytoid dendritic cells in the tumor microenvironment: immune targets for glioma therapeutics. Neoplasia. 2012;14(8):757–770. doi: 10.1593/neo.12794. 22952428 PMC3431182

[59] Lin YJ, Wei KC, Chen PY, Lim M, Hwang TL. Roles of neutrophils in glioma and brain metastases. Front Immunol. 2021;12:701383. doi: 10.3389/fimmu.2021.701383. 34484197 PMC8411705

