# Supplementary material for: Human microbiota influence the immune cell composition and gene expression in the tumor environment of a murine model of glioma
Source: Gut Microbes. 2025 May 30;17(1):2508432. doi: 10.1080/19490976.2025.2508432 (PMC12128662; doi:10.1080/19490976.2025.2508432)
Supplement: Green Supplementary Files.docx [file KGMI_A_2508432_SM4936.docx]

**
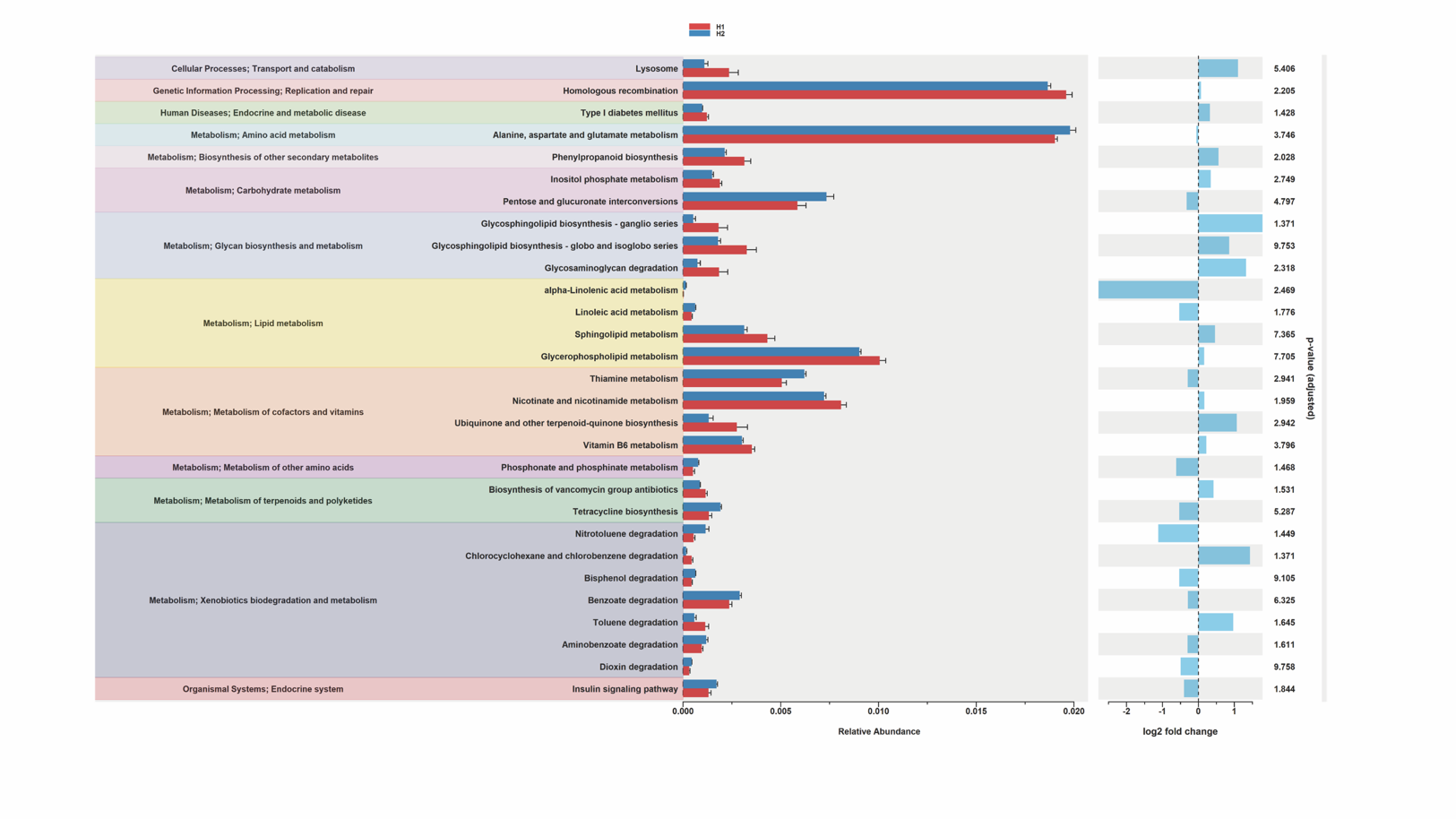
**

**Supplementary Figure 1.** Functional differences between HuM1 and HuM2 microbiota.

**
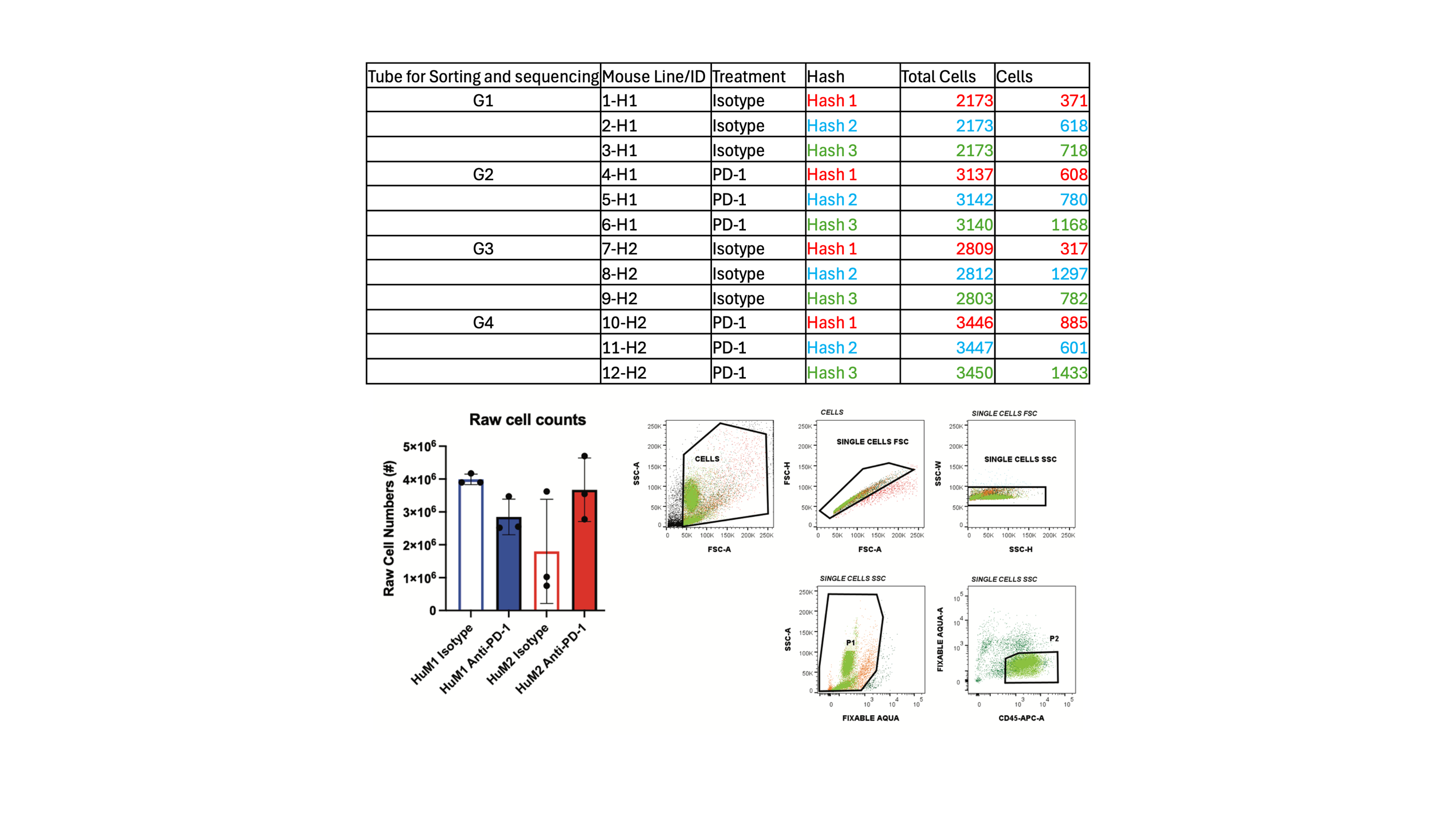
Supplementary Figure 2.** Schematic of single cell RNA sequencing experiment, including cell counts and gating analysis for each individual sample.

**
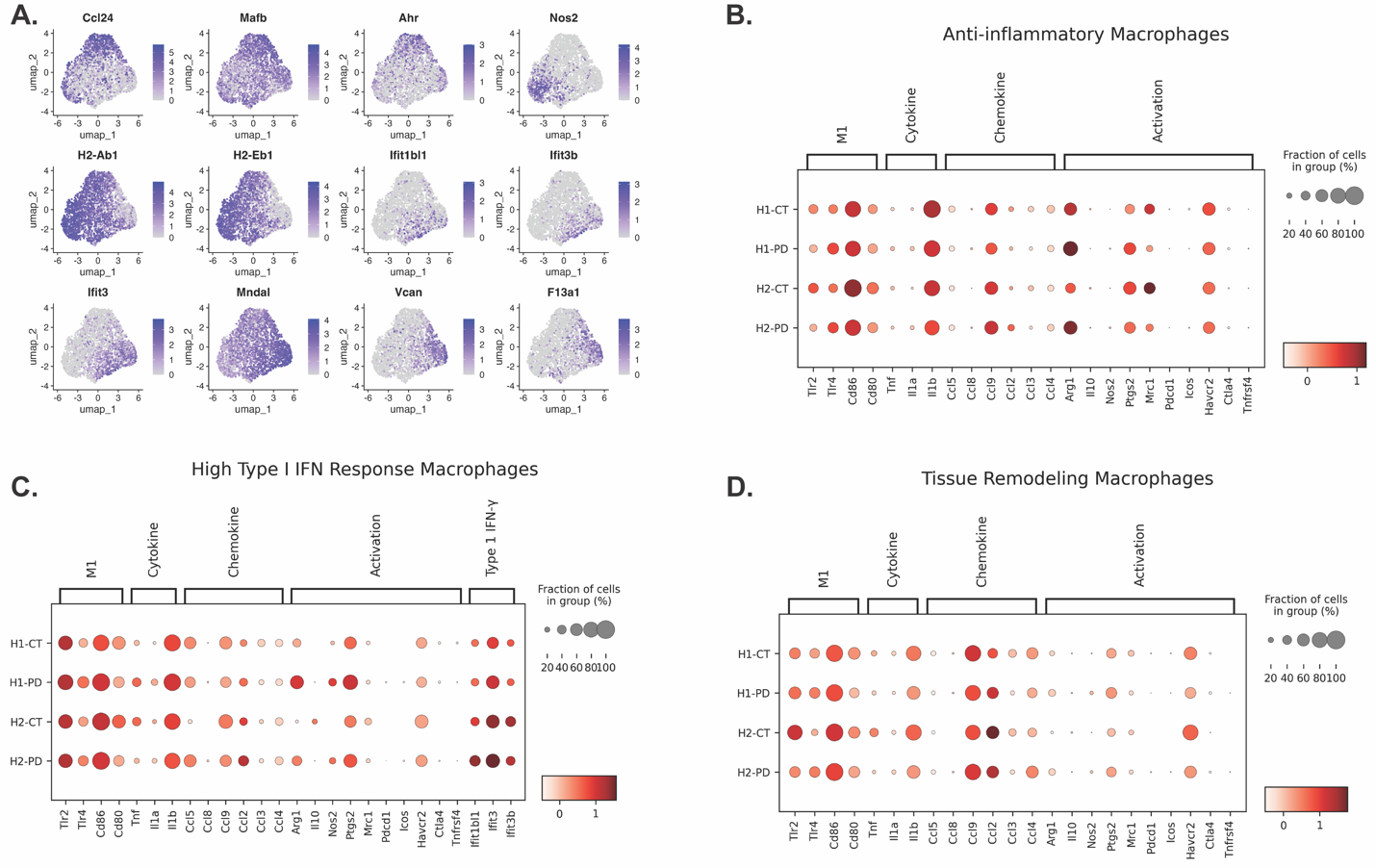
Supplementary Figure 3.** Single cell analysis of MM1 cell population. (**A**) Feature plot of marker genes. (**B**) Anti-inflammatory Macrophages dot plot. (**C**) High Type I IFN Response Macrophages dot plot. (**D**) Tissue Remodeling Macrophages dot plot.

**
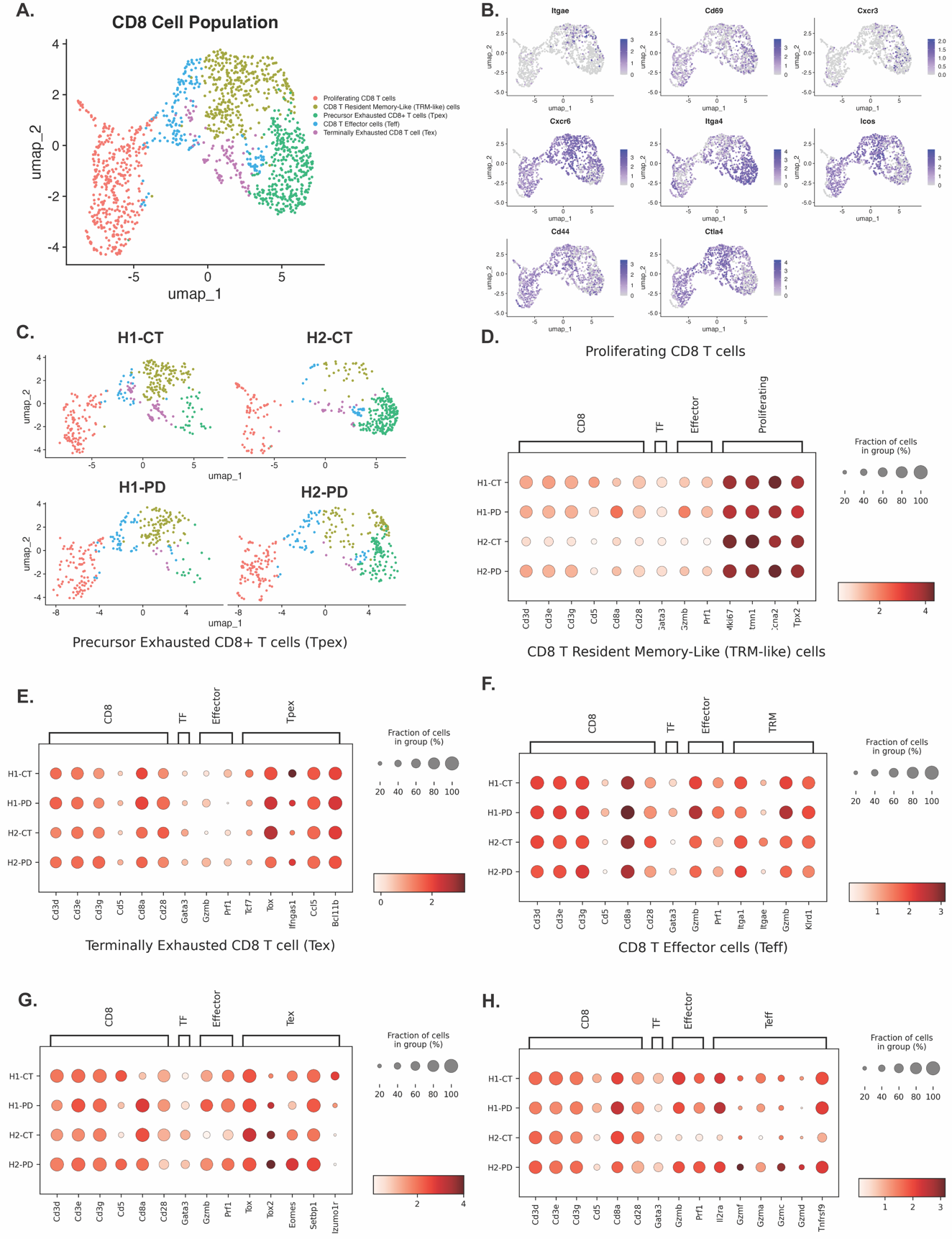
**

**Supplementary Figure 4.** Single cell analysis of CD8 cell population. (**A**) UMAP of CD8 population. (**B**) Feature plot of marker genes. (**C**) UMAP separated by sample ID. (**D**) Proliferating CD8 T cells dot plot. (**E**) Precursor Exhausted CD8+ T cells (Tpex) dot plot. (**F**) CD8 T Resident Memory-Like (TRM-like) cells. (**G**) Terminally Exhausted CD8 T cell (Tex). (**H**) CD8 T Effector cells (Teff).

**
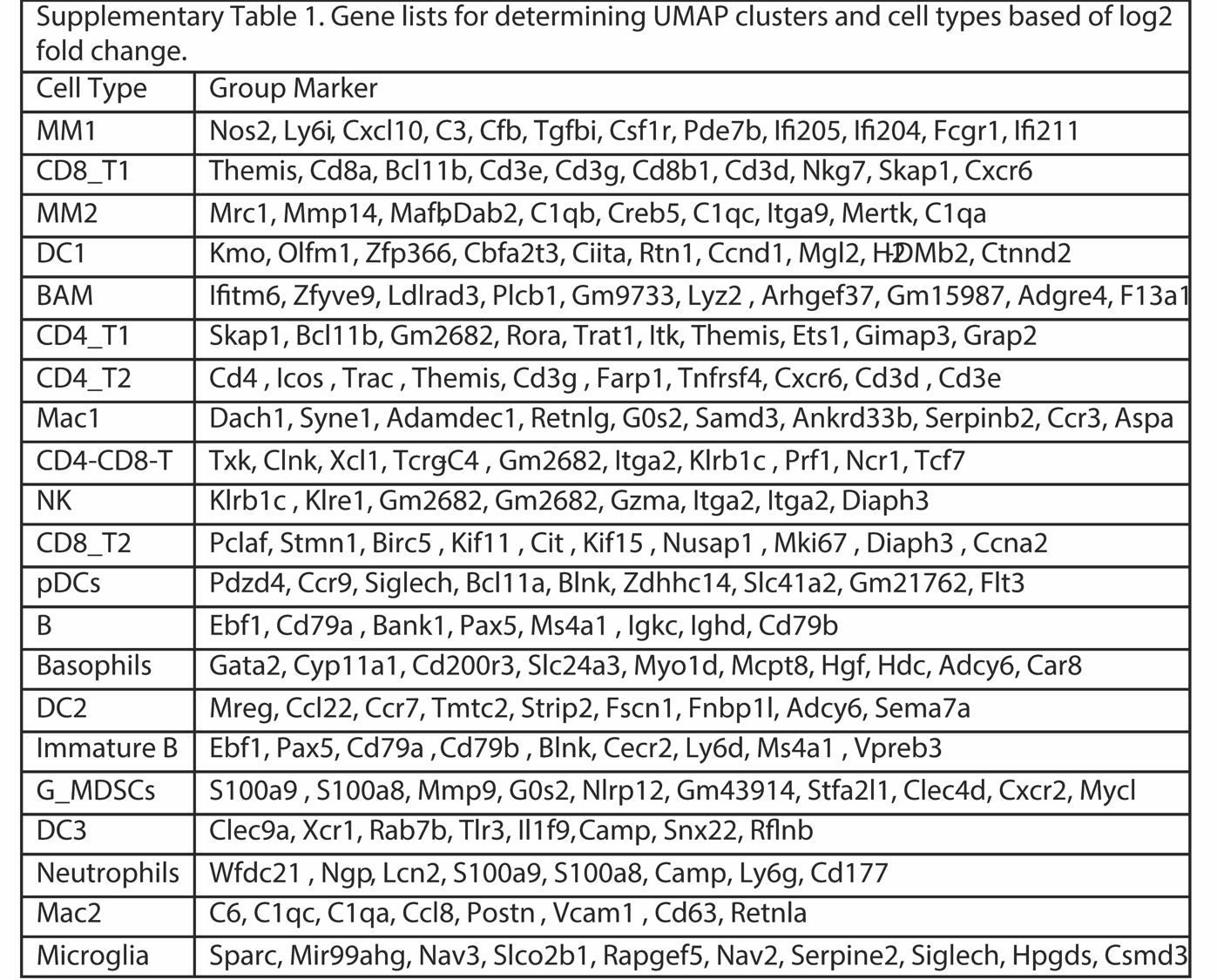
**
